# Gender effect in human–machine communication: a neurophysiological study

**DOI:** 10.3389/fnhum.2024.1376221

**Published:** 2024-07-10

**Authors:** Yi Ding, Ran Guo, Wei Lyu, Wengang Zhang

**Affiliations:** School of Economics and Management, Anhui Polytechnic University, Wuhu, China

**Keywords:** virtual chatbots, gender difference, usage intention, ERPs, human–robot interaction

## Abstract

**Purpose:**

This study aimed to investigate the neural mechanism by which virtual chatbots' gender might influence users' usage intention and gender differences in human–machine communication.

**Approach:**

Event-related potentials (ERPs) and subjective questionnaire methods were used to explore the usage intention of virtual chatbots, and statistical analysis was conducted through repeated measures ANOVA.

**Results/findings:**

The findings of ERPs revealed that female virtual chatbots, compared to male virtual chatbots, evoked a larger amplitude of P100 and P200, implying a greater allocation of attentional resources toward female virtual chatbots. Considering participants' gender, the gender factors of virtual chatbots continued to influence N100, P100, and P200. Specifically, among female participants, female virtual chatbots induced a larger P100 and P200 amplitude than male virtual chatbots, indicating that female participants exhibited more attentional resources and positive emotions toward same-gender chatbots. Conversely, among male participants, male virtual chatbots induced a larger N100 amplitude than female virtual chatbots, indicating that male participants allocated more attentional resources toward male virtual chatbots. The results of the subjective questionnaire showed that regardless of participants' gender, users have a larger usage intention toward female virtual chatbots than male virtual chatbots.

**Value:**

Our findings could provide designers with neurophysiological insights into designing better virtual chatbots that cater to users' psychological needs.

## 1 Introduction

Virtual chatbots, which are machine conversation systems equipped with chat interfaces, facilitate natural language interactions between humans and machines (Shawar and Atwell, 2005). With the application of more advanced and intelligent interfaces, chatbots enable users to engage in real-time communication and interaction with service providers (Xu et al., 2020; Adam et al., 2021). Since the invention of the world's first chatbot, ELIZA, by Joseph Weizenbaum in the 1960's, chatbots have revolutionized our modes of communication and found wide-ranging applications in fields, such as healthcare, e-commerce, retail, insurance, and customer service (Kasilingam, 2020; Mogaji et al., 2021). In the wake of the COVID-19 pandemic, the emergence of virtual chatbots has aided the logistics and supply chain services industry in maintaining communication with customers and providing uninterrupted services (Viola et al., 2021). This development has also propelled significant growth in the global virtual chatbot market. According to a report by Statista (2021), the market value of virtual chatbots is projected to reach $6.83 billion.

The constituent elements of a virtual chatbot include human figures (i.e., visual clues), names (i.e., identity cues), and chat dialogues (i.e., conversation clues; Go and Sundar, 2019). Among these, visual clues can affect consumers' intentions and decisions (Filieri et al., 2021). Particularly, gender, as part of the visual cues of virtual chatbots, significantly influences users' initial impressions, attitudes, and willingness to interact with chatbots (Calvo-Barajas et al., 2020; Zogaj et al., 2023). However, the role of users' own gender in this perception and interaction has also garnered attention. Studies have indicated that the design and presentation of gender can evoke emotional responses from users, thereby influencing their usage experience and satisfaction. Moreover, users' own gender can also influence their responses to gender cues presented by chatbots.

Therefore, this study aims to explore not only the impact of the presentation of gender on the design of virtual chatbots but also its interplay with users' gender. We can provide valuable insights into designing more humanized and effective chatbots by understanding the role of gender in human–machine communications.

### 1.1 The effects of virtual chatbots' gender

Gender is a central dimension of individuals' self-concept and identity, making it a key human attribute that significantly influences how people form connections with others (Freimuth and Hornstein, 1982). Gender-related social cues can minimize the need for extra information-seeking during interactions (Tay et al., 2014). The gender of robots fosters a sense of shared understanding between users and robots, leading to more natural and intuitive human–robot interactions (Powers et al., 2005; Eyssel and Hegel, 2012). Some studies have shown that the gender of chatbots may affect consumer behavior (Seo, 2022; Zogaj et al., 2023).

When exploring the role of gender in human–machine interaction, one aspect that cannot be overlooked is gender stereotypes. Individuals generally believe that women are more suitable for taking care of children or older adults and that male surgeons are more capable than female surgeons (Eagly, 2013; Ashton-James et al., 2019). This phenomenon reflects the existence of gender stereotypes. In reality, gender stereotypes are an enduring concept that emphasizes social consequences arising from gender cues (Tay et al., 2014). The study by Master et al. (2021) demonstrated gender stereotypes, indicating that girls are less interested than boys in computer science and engineering. These stereotypes can even extend to non-human agents (Tay et al., 2014). Apple's Siri and Amazon's Alexa typically use female voices (Chin and Robison, 2023), and Samsung's Sam is presented with a female avatar. Abdulquadri et al.'s (2021) also found that chatbots in emerging market banks are frequently branded and associated with female gender identification. On the contrary, Behrens et al. (2018) conducted a limited study, which indicated a tendency to trust male robots more than female robots. Likewise, Ahn et al. (2022) found that participants give higher competence scores to male rather than female AI agents. After reviewing existing studies on the effects of gender on human–machine communication, the perception of robots' gender remains controversial. Thus, this study aims to further explore how the gender of virtual chatbots influences users' usage intention.

This research not only discusses the impact of gender on virtual robots but also explores the influence of human gender differences on human–machine communication. Previous research has shown that human gender differences can influence perceptual experiences of things (Gefen and Straub, 1997; Qu and Guo, 2019; Denden et al., 2021). For instance, in a study by Nissen and Krampe (2021), an examination was carried out regarding how users consciously and unconsciously (neural) evaluate e-commerce websites. They found that unconscious effects influence gender-related differences in the perception of e-commerce websites. Huang and Mou (2021) discovered that, within current online travel agency websites, women exhibit more usability requirements than men. Relevant research frequently cites the similarity-attraction paradigm, which suggests that as the similarity to a target increases (i.e., similar attitudes, personality traits, or other attributes), the target's attractiveness also increases (Byrne, 1997). This study explores how men and women treat the gender of virtual chatbots in human–machine interaction and also delves into the gender preferences of virtual chatbots among different gender users.

### 1.2 Event-related potentials' method of revealing usage intentions of virtual chatbots' gender

Currently, research on the relationship between the gender of virtual chatbots and users' usage intentions is limited. Most studies evaluating users' intentions regarding the gender of virtual chatbots rely on questionnaires and interviews, which may not completely capture users' true emotions and are susceptible to various influencing factors. The intention to use, as a latent psychological activity, is difficult to articulate verbally (Ding et al., 2016), while cognition and emotion, as products of brain neural activity (Kim et al., 2022), play an important role in usage intentions (Kang et al., 2015). Therefore, event-related potential (ERP) methods are needed to measure users' intrinsic intentions, including unconscious formations.

ERPs, arising from postsynaptic potentials during neurotransmission, travel passively through the brain and skull to the scalp, thereby contributing to a broader electroencephalogram (EEG; Luck et al., 2000). The EEG can measure the neurophysiological data of users experiencing the information system objectively and in real time (Liu et al., 2022). ERPs offer insights into participants' brain responses to certain cognitive events and, ultimately, into their psychological activities (Luck, 2014; Sun et al., 2022). Therefore, they can be employed to investigate neural activities related to virtual chatbots. Research has shown that some ERP components can effectively increase individuals' attentional resources and emotional arousal (Ding et al., 2016; Liu et al., 2022). Current ERP research on attention is primarily focused on three key components: N100, P100, and P200 (Luck et al., 2000; Ding et al., 2016; Cao et al., 2021).

#### 1.2.1 The N100 component

The N100 component, as a crucial constituent of ERPs, peaks at ~100 ms post-stimulus presentation and manifests as a negative-going potential (Li et al., 2022). It is related not only to physical features in the reflection of people's attention allocation at an early stage (Luck et al., 2000) but also to attractiveness in the reflection of the capacity of stimuli to attract and maintain the participant's attention (Carretié et al., 2004). Stimuli perceived to have high attractiveness evoked an increased amplitude of N100 (Righi et al., 2014). Many previous studies have reported that N100 reflects attention allocation and attractiveness (Luck et al., 2000; Li et al., 2022; Liu et al., 2022). For example, Liu et al. investigated the impact of users' first impressions of websites on their subsequent behaviors and attitudes, utilizing ERP techniques to analyze users' evaluative processing. The study found that webpages higher in complexity and order evoked larger N100 amplitudes than those that were lower in complexity and order (Liu et al., 2022). Guo et al. examined visual attention toward humanoid robot appearances and observed that users devoted greater attentional resources to their preferred robots than to non-preferred ones (Guo et al., 2022).

#### 1.2.2 The P100 component

The P100 component (peaking around 90–100 ms post-stimulus presentation), as an early ERP, exhibits sensitivity to attention allocation (Liu et al., 2022). When more attention is directed to a visual stimulus, the amplitude of the P100 component increases, providing a direct indicator of attention (Smith et al., 2003), and it is typically related to physical stimulus characteristics (Perri et al., 2019). The role of P100 in reflecting attention capture has been widely reported in previous research (Perri et al., 2019; Yen and Chiang, 2021). Yen and Chiang used ERPs to explore the relationship between trust and purchase intention in the context of chatbots (Yen and Chiang, 2021). In addition, in their study on the attention allocated to app icons, Liu et al. utilized the early P100 component and found that complex icons elicited a higher amplitude of P100 than simple icons (Liu et al., 2024).

#### 1.2.3 The P200 component

P200, another positive-going potential that peaks around 200 ms post-stimulus presentation, is associated with the initial exogenous “attention capture” of the affective content of a stimulus (Carretié, 2014). Stimuli arousing positive or negative feelings elicited an increased P200 amplitude (Carretié et al., 2004; Liu et al., 2022). As the most conspicuous and widely used “attention” ERP, P200 was found in a number of attention-related studies (Carretié et al., 2004; Liu et al., 2022; Wang et al., 2023). For instance, Wang et al. (2023) used ERP techniques to explore consumers' emotional experiences and consumer trust when interacting with chatbots (vs. humans). The results revealed that the amplitudes of P200 were larger for chatbots than for humans. Guo et al. (2022) utilized 20 humanoid robot pictures as experiment stimuli to investigate users' preference for the appearance of humanoid robots. The research indicated that, in the early stage, the preferred humanoid robot appearances elicited larger P200 amplitudes than the non-preferred appearances.

Existing literature provides evidence for effectively applying ERPs, particularly N100, P100, and P200, in the research of the neural time course of attention to different stimuli. Hence, this study will first examine differences in the allocation of attentional resources among participants toward virtual chatbots of different genders. Second, it will investigate how the gender of the participants themselves contributes to these differences in attentional resources allocated to virtual chatbots of different genders.

### 1.3 Research hypotheses

Female roles in the service domain are more popular and predominant (Seo, 2022). However, the impact of gender on users' attention and willingness to use virtual service agents in human–machine communication remains unclear. The literature on gender stereotypes suggests that gender can serve as a direct categorization cue, influencing users' perceptions during service encounters (Macrae and Martin, 2007). Gendered service robots can evoke emotional responses such as attractiveness and likability (Macrae and Martin, 2007). The attractiveness bias effect suggests that perceived attractiveness tends to elicit positive evaluations due to attractiveness biases. Moreover, this effect is amplified when service roles are designated as female (Hosoda et al., 2003). Indeed, the research findings by Stroessner and Benitez on gendered humanoid robots support this notion, which revealed that female humanoid robots elicited more positive evaluations and a greater desire for engagement among consumers (Stroessner and Benitez, 2019). Therefore, the research hypothesizes the following:

H1: Participants exhibit a higher willingness to use female virtual chatbots than male virtual chatbots; additionally, female virtual chatbots elicit greater N100, P100, and P200 amplitudes in the participants than male virtual chatbots.

Given the influence of gender congruence on interpersonal relationships in fields such as human resource management and organizational behavior (Crijns et al., 2017), the similarity-attraction paradigm (Byrne, 1997) posits that attraction toward a target increases with greater similarity to the target, such as similarity in attitudes, personality traits, or other attributes. Individuals find it easier to engage with robots when they possess gender and personality characteristics (Vecchio and Bullis, 2001). In the field of human–machine communication, existing research (Tay et al., 2014) suggests that individuals are more likely to accept robots that align with their own gender and personality traits. This implies that as similarity increases, intention to use and accept robots in social contexts also increases. Therefore, this study hypothesizes the following:

H2: Participants may be more inclined to use virtual chatbots of the same gender. Specifically, for female participants, there is a greater inclination to use female virtual chatbots than male virtual chatbots; conversely, for male participants, there is a greater inclination to use male virtual chatbots than female virtual chatbots.

Gender congruence may influence participants' attention. Previous research has shown that female participants are more likely to accept female robots than male participants; male participants show a higher acceptance level for male robots than for female robots (Nass et al., 1997). This finding aligns with the similarity-attraction paradigm (Byrne, 1997) and suggests that gender congruence can lead to users' positive perceptions of robots, psychological closeness, and potentially further increase attention allocation to robots (Eyssel et al., 2012). Given that attention-related EEG indicators such as N100, P100, and P200 can reflect users' attention allocation, this study hypothesizes the following:

H3: For female participants, female virtual chatbots evoke larger N100, P100, and P200 amplitudes than male virtual chatbots; conversely, for male participants, male virtual chatbots evoked larger N100, P100, and P200 amplitudes than female virtual chatbots.

## 2 Research measures

### 2.1 Participants

A prior calculation was conducted to determine the required sample size using G^*^Power3.1 (Erdfelder et al., 1996): a minimum sample size of 12 was needed to detect a large effect size (f = 0.4) with a recommended statistical power β of 95% and an error probability α of 0.05. For the ERP experiment, we recruited 33 participants (16 female and 17 male participants) via WeChat, excluding two female participants due to power failure. Hence, the final analysis covered 31 participants (17 male and 14 female participants). They were all students from AHPU, of Han ethnicity, aged 19–28 years (M= 21.58, SD = 2.28); furthermore, they had normal/corrected vision, were right-handed, and remained medication-free for a week. Before the experiment, they ensured that they had sufficient sleep, had no neurological/mental disorders, and signed an informed consent form. They received RMB 70 as remuneration. The study was approved by the Ethics Committee of the Institute of Neuroscience and Cognitive Psychology at AHPU.

### 2.2 Stimuli

A stimulus set consisting of six non-target stimulus images and two images of flowers as target pictures was assembled. The non-target stimulus images were sourced from the Vision China website (https://www.vcg.com), with three images featuring men and three images featuring women. We utilized Adobe Photoshop 2018 outlining and contouring tools to accentuate the lines and features of the characters in the images to achieve a humanoid robot effect. Subsequently, we adjusted the color, contrast, brightness, and saturation of the images in Photoshop to accentuate the mechanical feel. Next, while processing the characters' facial features, we conducted detailed refinement to make them appear more robot-like. Finally, we compared and fine-tuned the processed images with the current highly humanoid robots, Geminoid H1-4 and Kodomoroid, to make them appear closer to the target effect. All images were designed to have dimensions of 1920^*^1150 pixels.

### 2.3 Procedure

**Figure 1:**
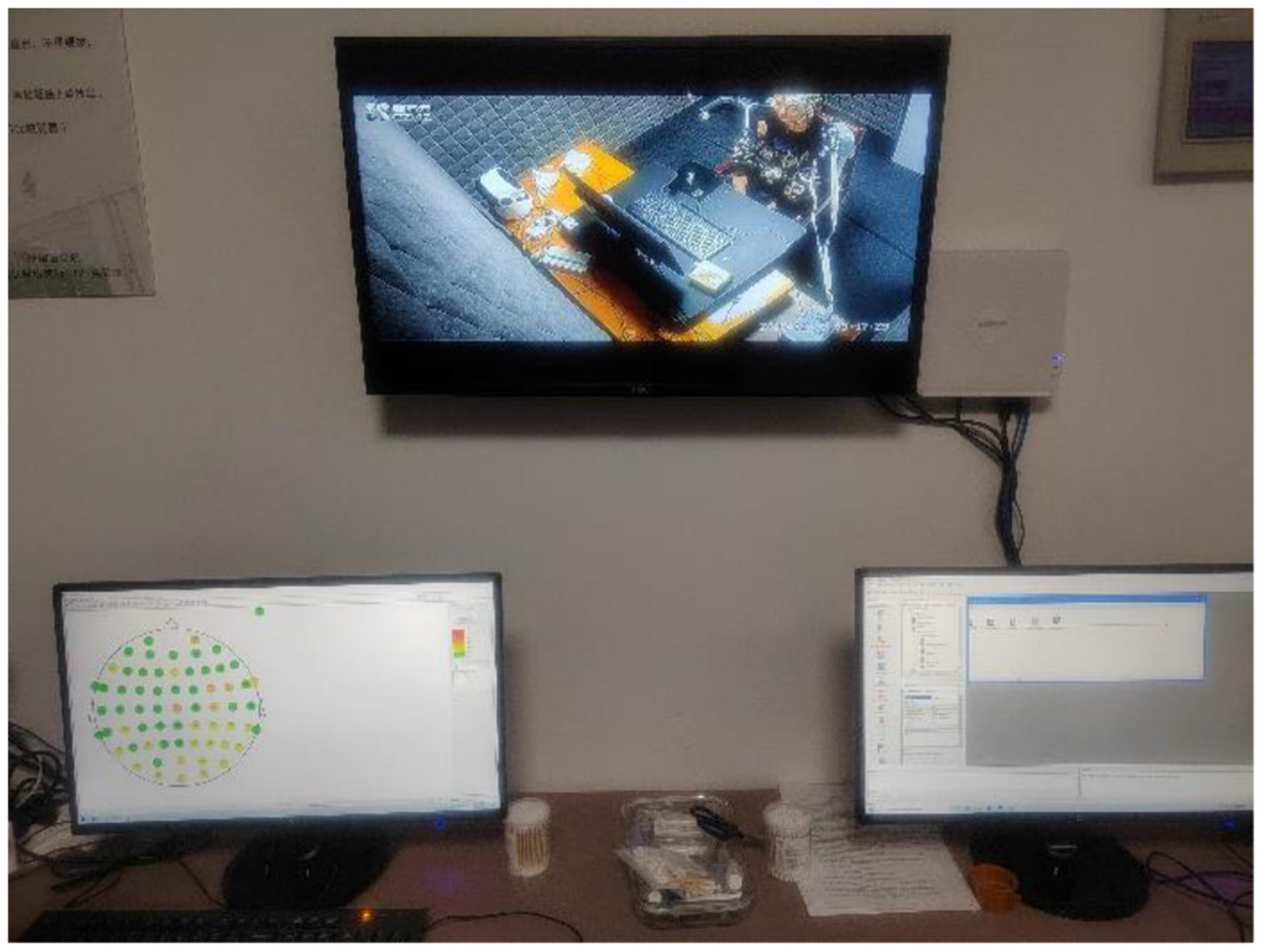
ERP laboratory environment.

This experiment was conducted in a professional ERP laboratory divided into a preparation room and an observation control room (Figure 1). The preparation room provided a suitable environment with sound insulation, suitable light, temperature and humidity, and minimal external interference. The observation control room allowed the experimenter to control the experiment process and observe any abnormal conditions in the participants. During the preparation phase, participants were instructed to wash and blow-dry their hair, which aimed to reduce impedance between the electrode and the scalp to ensure the accuracy and reliability of the EEG signal acquisition. They then entered the preparation room and sat on a chair ~80 cm away from the computer screen, with their gaze fixed on the center of the screen. Following the international 10–20 system principles, the Cz electrode site was determined by the intersection of the line connecting bilateral earlobes and the line from the nasion to the inion. Subsequently, an appropriate electrode cap was worn. After the preparation was completed, the participants were informed about the instructions of the experiment.

At the beginning of the experiment, participants were informed that it was a scenario-based task. The scenario was as follows: Assuming your good friend's birthday is approaching, you want to buy a short-sleeved shirt as a birthday gift. However, you do not know your friend's clothing size; you only know their height and weight. Therefore, you open Taobao and browse a short-sleeved shirt design that you like. To obtain sizing information, you decide to consult with two types of virtual chatbots, William or Lily. Lily, a female virtual chatbot, exhibits pronounced feminine facial characteristics that mimic human features, while William, a male counterpart, possesses distinct male facial traits that emulate those of a real human. In the following questionnaire and EEG experiment, all male virtual chatbots are named William, and all female virtual chatbots are named Lily.

**Figure 2:**
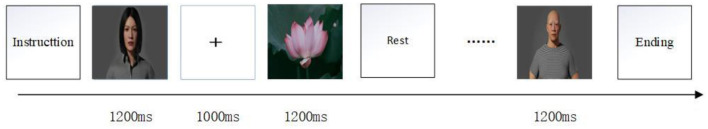
Flowchart for the ERP experiment with virtual chatbots.

The EEG experiment was programmed and demonstrated by E-prime 3.0. The experiment utilized an oddball paradigm, and the stimulus materials included a virtual chatbot, a non-target stimulus (180 times), and a flower target stimulus (60 times). The stimuli were presented randomly, with each stimulus presented 30 times for a duration of 1,200 ms, with a “+” fixation point shown in the center of the screen for 500 ms between the two stimuli. There was one rest period in the middle of the experiment (Figure 2). Participants were instructed to remember the occurrence of all the target stimuli.

At the end of the experiment, participants were asked to rate their intention to use the virtual chatbots for six non-target stimuli. The usage intention was evaluated using three questions based on Agarwal and Karahanna's (2000) work: “I plan to use the virtual chatbots,” “I intend to continue using the virtual chatbots,” and “I expect to use the virtual chatbots in the future.” The usage intention was rated on a 5-point Likert scale, with “1” meaning strongly disagree and “5” meaning strongly agree. After filling out the scale, the entire experiment concluded. The overall task required ~40 min to complete, comprising a preparatory phase of 25 min, ~10 min for the ERP task, and 5 min for the completion of the questionnaire.

### 2.4 EEG recording and analysis

EEG data were recorded using a Brain actiCHamp amplifier (Brain Products GmbH, Munich, Germany) and a cap with 64 g/AgCl electrodes following the international 10–20 system. Cz was used as the reference electrode. The EEG data were bandpass filtered with a range of 0.05–70 Hz and continuously sampled at 1,000 Hz. The impedance between the scalp and electrodes was kept below 5 KΩ.

Offline EEG data were analyzed using EEGLAB (version 2019.0), an open-source toolbox developed by Delorme and Makeig (2004). The reference electrode Cz was replaced with TP9 and TP10, and the sampling rate was reduced to 500 Hz. The bandpass filter was 0.1–30 Hz. Eye movement artifacts, muscle artifacts, and other artifacts were manually removed using independent component analysis. EEG signal segments exceeding 75 μV were automatically removed, and bad channels identified visually were rejected. The rejected channels were then reinserted using a spherical interpolation method. Then, EEG signals were computed using EEG epochs that started from 200 ms before the onset of the target stimulus to 1,000 ms after the stimulus' onset. Moreover, each epoch was baseline corrected using the signal during 200 ms, which preceded the onset of the stimulus. Finally, EEG signal values related to the gender of the virtual chatbots were superimposed and averaged to generate grand-averaged ERP waveforms and scalp topographies.

### 2.5 Statistical analysis

Mean amplitude and usage intention values for ERPs and subjective evaluation data were subjected to repeated measures ANOVA. A 2 (virtual chatbot gender: male and female) × 3 (brain region: central-parietal, parietal, parietal-occipital, and occipital) repeated measures ANOVA was utilized in this study. Additionally, to investigate the influence of participants' gender on ERPs and usage intention data, we analyzed male/female participants on two factors: virtual chatbot gender and brain sites, respectively, using repeated measures ANOVA for the mean amplitude and usage intention. We used the Greenhouse–Geisser correction for any violation of the sphericity assumption (uncorrected df and corrected *p*-values were reported). The alpha level was fixed at 0.05. All statistical analyses were conducted using SPSS22.0.

## 3 Result

### 3.1 Subjective questionnaire

The reliability and validity of the scale were tested using SPSS 22.0. The results showed that Cronbach's alpha was 0.855, indicating very good internal consistency. The scale's validity was assessed by performing exploratory factor analysis. After the extraction of factors by using Promax rotation, the Kaiser–Meyer–Olkin (KMO) value (KMO = 0.608) was obtained. Bartlett's test of sphericity was extremely significant, suggesting the suitability of the data for factorization.

A repeated measures ANOVA was conducted to test for differences in usage intention among virtual chatbots and participants' gender; the results suggested a significant main effect of virtual chatbots' gender [*F*_(1.000, 32.000)_ = 14.827, *p* = 0.001, partial-η^2^ = 0.317]. There was a significant main effect of virtual chatbots' gender for female participants [*F*_(1.000, 15.000)_ = 7.737, *p* = 0.014, partial-η^2^ = 0.340]. The main effect of virtual chatbots was significant for male participants [*F*_(1.000, 16.000)_ = 7.101, *p* = 0.017, partial-η^2^ = 0.307]. The pair comparison results revealed that female virtual chatbots evoked larger user usage intentions than male virtual chatbots [participants: *p* = 0.001; female participants: *p* = 0.014; male participants: *p* = 0.017].

### 3.2 Electrophysiology

**Figure 3:**
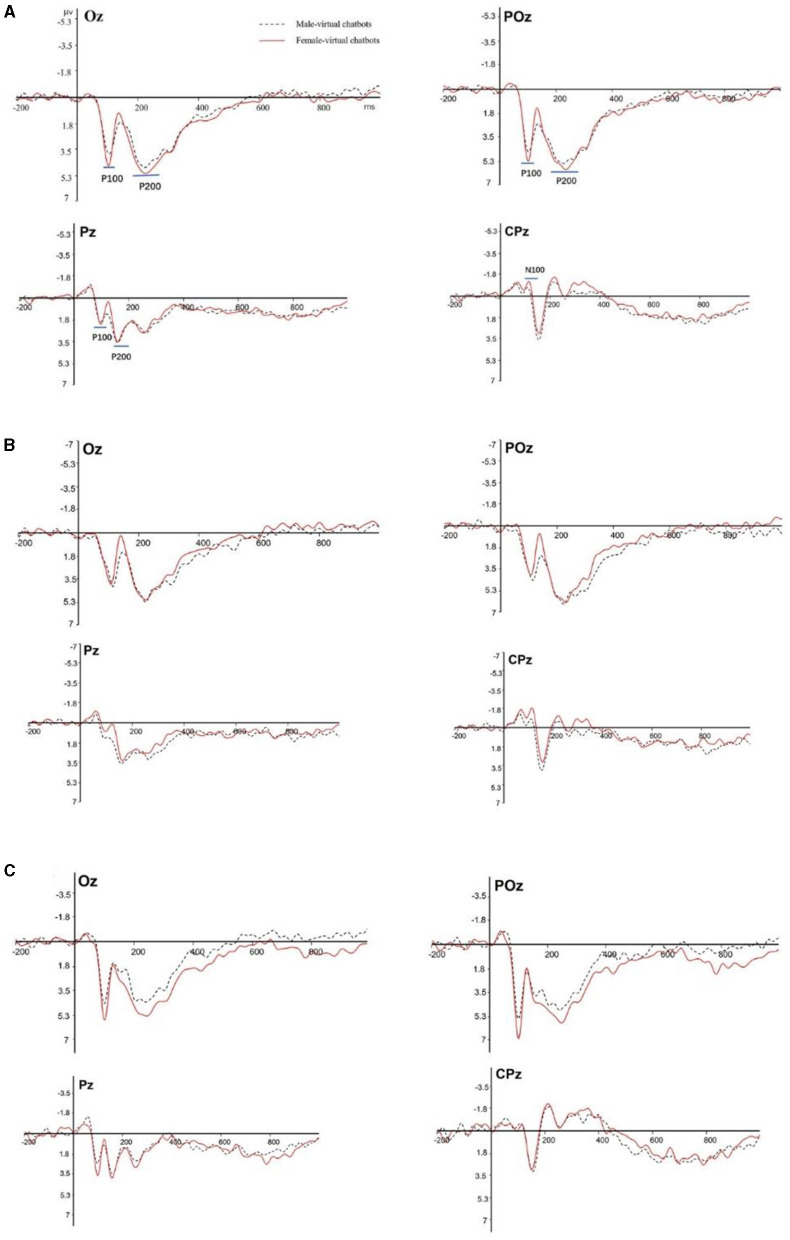
The grand-averaged waveform for virtual chatbots. **(A)** Shows the waveform without distinguishing the participants, **(B)** shows the waveform for female participants, and **(C)** shows the waveform for male participants.

**Figure 4:**
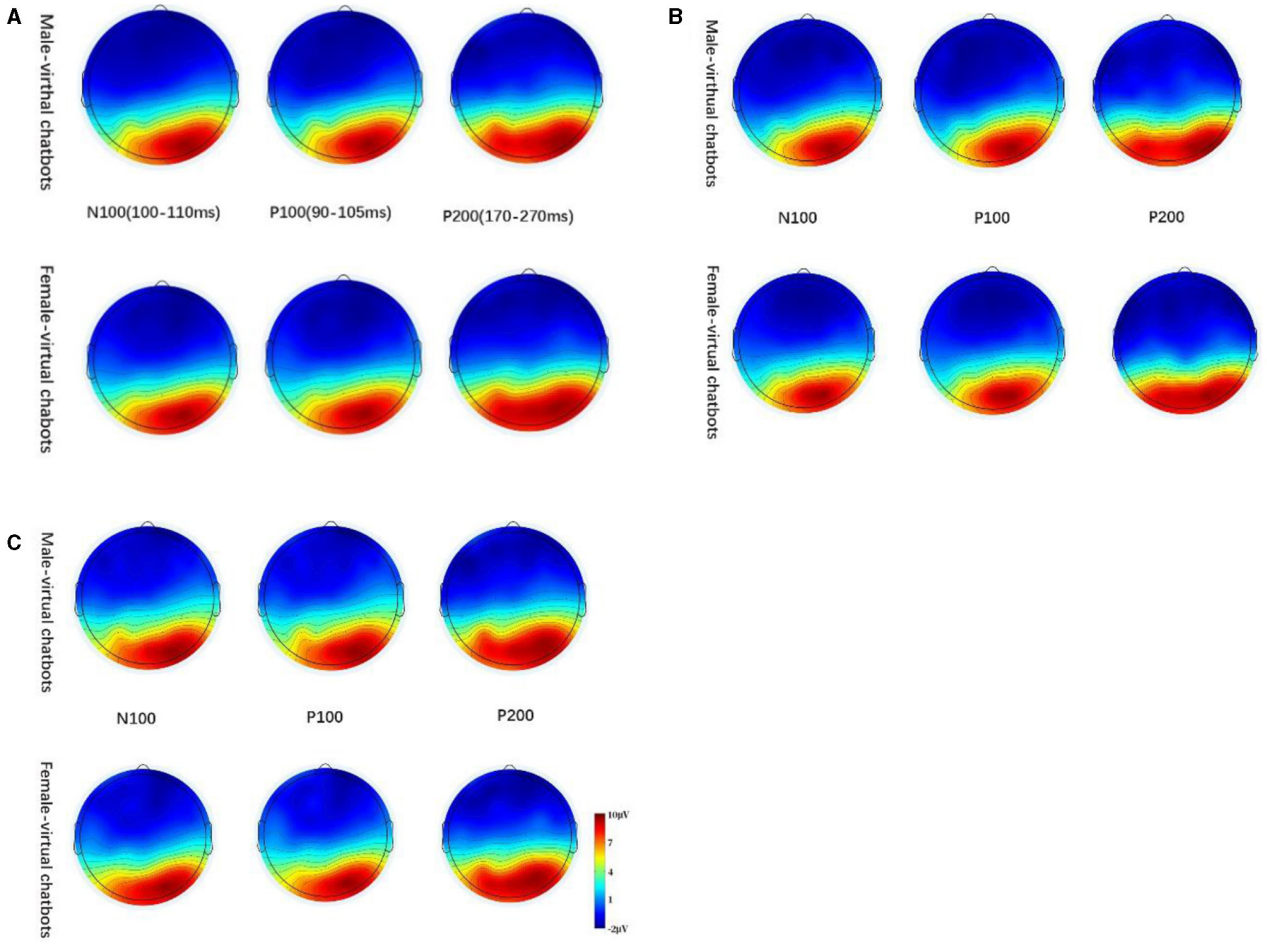
The scalp topographic maps of virtual chatbots. **(A)** Shows the scalp topographic maps without distinguishing the participants, **(B)** shows the scalp topographic maps for female participants, and **(C)** shows the scalp topographic maps for male participants.

Based on grand-averaged ERP waveforms (Figure 3), scalp topographic maps (Figure 4), and extant studies (Ding et al., 2016; Guo et al., 2022), we chose the central-parietal (CP1, CPZ, and CP2), parietal (P3, PZ, and P4), parietal-occipital (PO3, POZ, and PO4), and occipital (O1, OZ, and O2) sites for subsequent ERP analysis. As shown in Figure 3, we selected the P100 component in the time window of 90–105 ms in the parietal, parietal-occipital, and occipital sites. We chose the P200 component in the time window of 170–270 ms in the parietal, parietal-occipital, and occipital sites. We selected the N100 component in the time window of 100–110 ms in the central parietal site. The 12 electrodes were divided into four subgroups: a central-parietal group (CP1, CPZ, and CP2), a parietal group (P3, PZ, and P4), a parietal-occipital group (PO3, POZ, and PO4), and an occipital group (O1, OZ, and O2).

#### 3.2.1 N100

**Figure 5:**
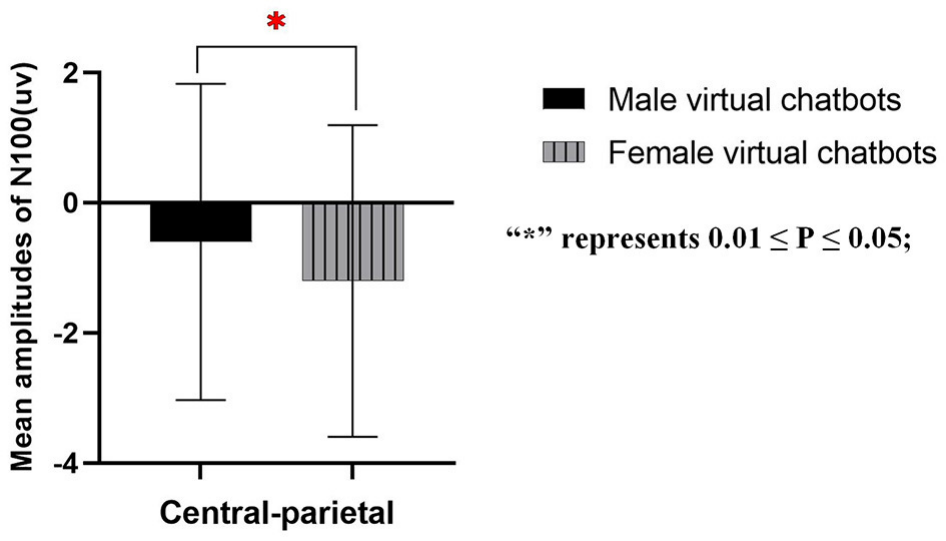
Mean amplitudes of N100 in the central-parietal area. “***” represents *P* ≤ 0.001; “**” represents 0.001 ≤ *P* ≤ 0.01; “*” represents 0.01 ≤ *P* ≤ 0.05; and “ns” represents non-significance.

Participants engaging with two different types of virtual chatbots exhibited varying perceptions of the neural activities attributed to the chatbots. The main effect of the brain sites was significant [central-parietal: *F*_(1.383, 42.869)_ = 9.437, *p* = 0.001, partial- η^2^ = 0.254]. There was no significant main effect of virtual chatbots' gender [central-parietal: *F*_(1.000, 31.000)_ = 0.124, *p* = 0.727, partial- η^2^ = 0.004] and no significant interaction effect between virtual chatbots' gender and brain sites [central-parietal: *F*_(1.124, 32.540)_ = 1.071, *p* = 0.336, partial- η^2^ = 0.033]. During the engagement between male participants and virtual chatbots, there was a significant main effect of virtual chatbots' gender [central-parietal: *F*_(1.000, 16.000)_ = 4.725, *p* = 0.045, partial- η^2^ = 0.228]. The pair comparison result showed that male virtual chatbots induced a larger amplitude of N100 than female virtual chatbots (as shown in Figure 5). During interactions between female participants and virtual chatbots, there was a significant main effect of brain sites [central-parietal: *F*_(2, 28)_ = 8.109, *p* = 0.002, partial- η^2^ = 0.367]. No significant effect was found on the others.

#### 3.2.2 P100

**Figure 6:**
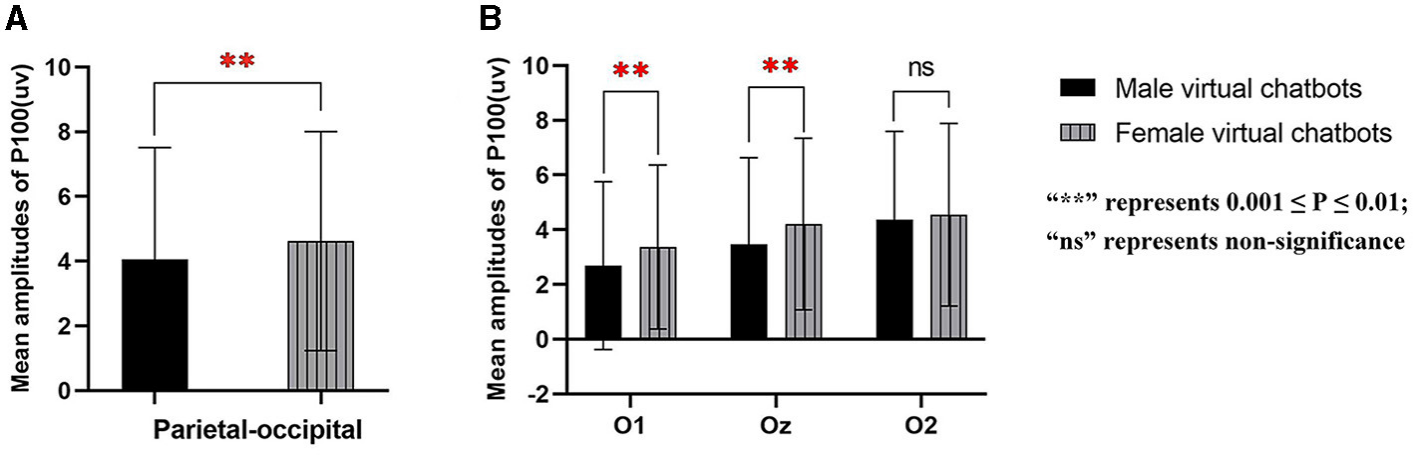
Mean amplitudes of P100 in parietal-occipital **(A)** and occipital areas **(B)**. ^**^represents 0.001 ≤ *P* ≤ 0.01.

There was a significant main effect of virtual chatbots' gender [parietal-occipital: *F*_(1.000, 31.000)_ = 4.809, *p* = 0.036, partial- η^2^ = 0.134] and brain sites [parietal: *F*_(1.461, 45.282)_ = 15.379, *p* < 0.001, partial- η^2^ = 0.332; parietal-occipital: *F*_(2, 62)_ = 31.039, *p* < 0.001, partial- η^2^ = 0.500]. There was no significant main effect of virtual chatbots' gender [parietal: *F*_(1.000, 31.000)_ = 0.385, *p* = 0.539, partial- η^2^ = 0.012] and no significant interaction effect between virtual chatbots' gender and brain sites [parietal: *F*_(1.440, 44.631)_ = 0.027, *p* = 0.936, partial- η^2^ = 0.001; parietal-occipital: *F*_(1.620, 50.221)_ = 0.062, *p* = 0.908, partial- η^2^ = 0.002]. The pair comparison result revealed that female virtual chatbots evoked a larger amplitude of P100 than male virtual chatbots [parietal-occipital: *p* = 0.036] (as shown in Figure 6A). For the occipital area, the interaction effect between virtual chatbots' gender and brain sites was significant [occipital: *F*_(2, 62)_ = 6.734, *p* = 0.002, partial- η^2^ = 0.178]. The simple effect showed that female virtual chatbots induced a significantly larger amplitude of P100 than male virtual chatbots [O1: *p* = 0.008; Oz: *p* = 0.008] (as shown in Figure 6B).

**Figure 7:**
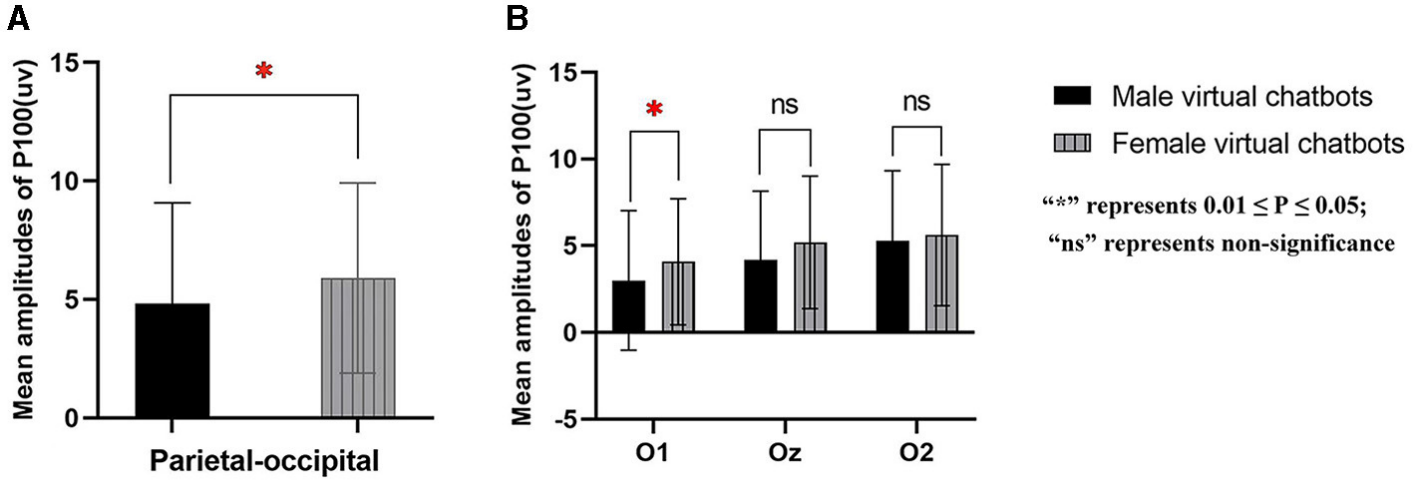
Mean amplitudes of P100 from female participants in parietal-occipital **(A)** and occipital areas **(B)**. ^*^represents 0.01 ≤ *P* ≤ 0.05.

In the interaction process between female participants and virtual chatbots, there was a significant main effect of virtual chatbots' gender [parietal-occipital: *F*_(1.000, 14.000)_ = 6.094, *p* = 0.027, partial- η^2^ = 0.303] and brain sites [parietal: *F*_(1.406, 19.680)_ = 6.987, *p* = 0.010, partial- η^2^ = 0.333; parietal-occipital: *F*_(2, 28)_ = 17.745, *p* < 0.001, partial- η^2^ = 0.559; occipital: *F*_(1.238, 17.335)_ = 15.361, *p* = 0.001, partial- η^2^ = 0.201]. The pair comparison result showed that female virtual chatbots evoked a larger amplitude than male virtual chatbots [parietal-occipital: *p* = 0.027] (as shown in Figure 7A). For the occipital area, the interaction effect between virtual chatbots' gender and brain sites was significant [*F*_(2, 28)_ = 5.292, *p* = 0.011, partial- η^2^ = 0.274]. The simple effect showed that female virtual chatbots induced a larger amplitude of P100 than male virtual chatbots [O1: *p* = 0.023] (as shown in Figure 7B).

#### 3.2.3 P200

**Figure 8:**
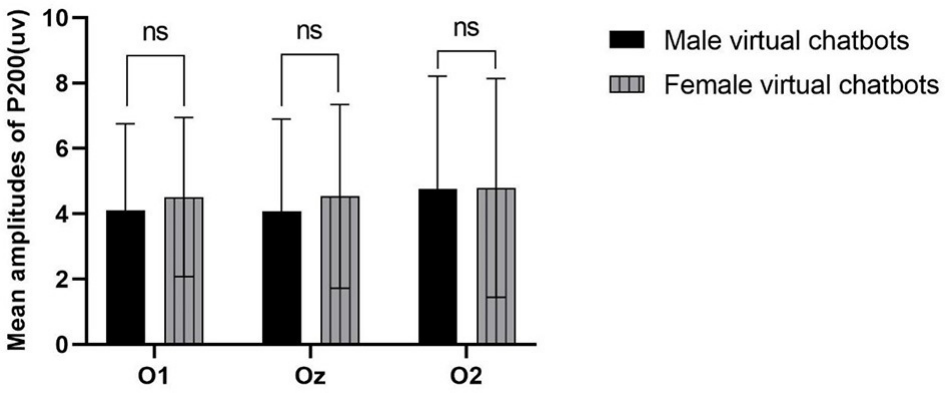
Mean amplitudes of P200 in the occipital area. ^*^represents 0.01 ≤ *P* ≤ 0.05.

There was no significant difference among other brain regions except for the occipital lobe area. However, we observed a significant interaction effect between virtual chatbots' gender and brain sites [occipital: *F*_(1.605, 49.769)_ = 3.471, *p* = 0.048, partial- η^2^ = 0.101]. The simple effect result indicated that female virtual chatbots evoked a larger amplitude than male virtual chatbots, and the difference was close to significant [O1: *p* = 0.136; Oz: *p* = 0.119] (as shown in Figure 8).

**Figure 9:**
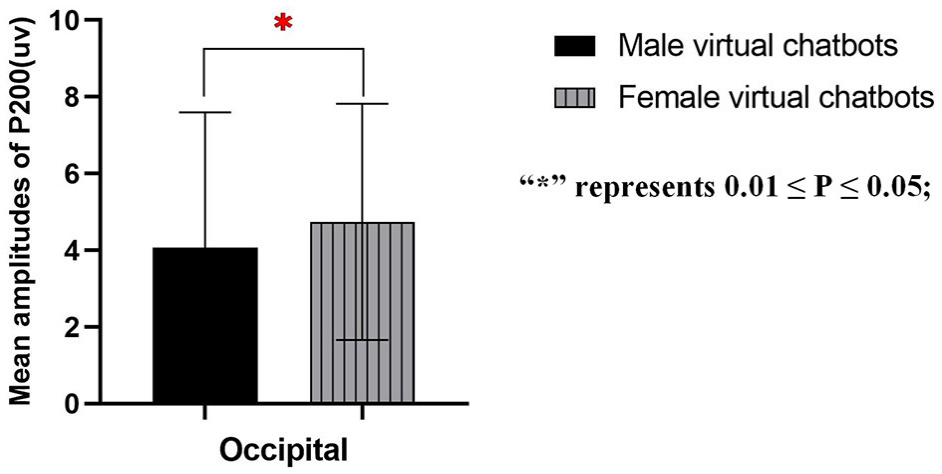
Mean amplitudes of P200 in female participants.

While female participants engaged with virtual chatbots, the main effect of virtual chatbots' gender^*^brain sites was significant [occipital: *F*_(1.000, 14.000)_ = 4.756, *p* = 0.047, partial- η^2^ = 0.254]. The pair comparison result indicated that in the occipital area, female virtual chatbots evoked larger P200 amplitudes than male virtual chatbots [*p* = 0.047] (as shown in Figure 9). When female participants interacted with virtual chatbots, no significant effect was found on other sites.

## 4 Discussion

### 4.1 The effect of virtual chatbots' gender on usage intention

In terms of usage intention, we found that virtual chatbots' gender significantly influenced users' usage intentions. The subjective evaluations indicated that, when the gender role of participants is not considered, people tend to prefer using female virtual chatbots. One plausible explanation is that virtual chatbots in the market are commonly associated with female voices, such as Apple's Siri and Amazon's Alexa (Fischer et al., 1997). This may lead to a more approachable quality associated with the female appearance, thereby increasing user acceptance and users' usage intentions. This result is consistent with gender stereotypes. Therefore, participants' intention to use female virtual chatbots was higher than their intention to use male virtual chatbots. The result supported H1.

When considering the gender factors of participants, the result suggested that among female participants, female virtual chatbots tend to have a higher usage intention than male virtual chatbots, whereas male participants tend to prefer using female virtual chatbots. The results from female participants are consistent with our hypothesis and the similarity-attraction paradigm (Byrne, 1997). A reasonable speculation is that women seek resonance and recognize the convenience of communication among the same gender. Therefore, female participants are more willing to use virtual chatbots of the same gender. Thus, H2 was confirmed. However, the results from male participants contradict our hypothesis and the similarity-attraction paradigm. One possible reason is that societal and cultural factors may influence the preferences of male participants, while individual differences may play a significant role among them. Therefore, male participants are more inclined to use female virtual chatbots. Thus, H2 was not supported.

### 4.2 The effect of virtual chatbots' gender on ERP components

Gender differences in cognition and their underlying brain mechanisms have attracted increasing attention ^(^Ramos-Loyo et al., 2022). This study used ERP techniques to analyze the evaluative process of virtual chatbots' gender. We found that regardless of whether participants' gender is considered a factor, the gender of virtual chatbots has an effect on the amplitudes of N100, P100, and P200.

#### 4.2.1 N100

N100 is sensitive to physical stimulus features and can reflect the allocation of attentional resources (Luck et al., 2000; Li et al., 2022). The results from male participants showed that, early in the time course, the gender of the virtual chatbots influenced the N100 amplitude, implying that the robot's gender attracted the user's attention. Specifically, male virtual chatbots elicited significantly larger N100 amplitudes than female virtual chatbots. This finding suggested that male participants would pay more attention to male virtual chatbots. Our results align with the similarity-attraction paradigm (Byrne, 1997), indicating that men are more inclined to engage with male virtual chatbots, thereby allocating greater attentional resources to them. This finding is consistent with those of previous studies. For instance, Bakar and McCann (2014) investigated the human–human gender congruity and found that gender congruity between supervisors and subordinates results in higher job satisfaction and commitment of subordinates. Similarly, Pitardi et al. research in the field of human–machine communication further confirmed that gender congruity significantly enhances the positive effects of communication (Pitardi et al., 2023). Thus, when male participants encountered male virtual chatbots, the larger N100 amplitude reflected their ability to induce a positive attentional resource, potentially due to the perceived congruity in gender. This result supported H3.

#### 4.2.2 P100

The present data displayed that early in the time course, female virtual chatbots enhanced a higher amplitude of P100 than male virtual chatbots in occipital and parietal-occipital areas, suggesting that the physical properties of virtual chatbots' gender can be detected. P100 is associated with the allocation of attentional resources in the early stage of processing visual stimuli (Nass et al., 1997). The results of this study suggested that female virtual chatbots required more attention than male virtual chatbots. One possible explanation for this observation could be that female virtual chatbots are perceived as more engaging or socially salient, thus capturing more attention at the early stages of visual processing. This could be attributed to cultural and societal biases that often associate femininity with warmth and social connection (Spence and Buckner, 2000). Therefore, the increased P100 amplitude may reflect a heightened sensitivity to, and the allocation of attention toward, female virtual chatbots. Thus, H1 was confirmed.

The results showed that among female participants, female virtual chatbots enhanced a higher amplitude of P100 in occipital and parietal-occipital areas than male virtual chatbots during the early processing stage. This suggested that female participants allocated more attention and cognitive resources to female virtual chatbots. One possible explanation is the concept of the similarity-attraction paradigm, where individuals tend to be attracted to and engage more with stimuli that are similar to themselves (Byrne, 1997). Our findings align with Eyssel et al.'s research (Eyssel et al., 2012), which revealed that participants developed more favorable impressions and reported greater psychological closeness when interacting with virtual chatbots of the same gender. This underscores the importance of similarity in fostering positive human–machine communication. Therefore, compared to male virtual chatbots, female participants would allocate more attention to female virtual chatbots. This result supported H3.

#### 4.2.3 P200

P200 is connected to research on emotion and attention arousal (Carretié et al., 2004; Liu et al., 2022). Our study revealed that female virtual chatbots induced a larger amplitude of P200 in occipital areas than male virtual chatbots. This suggested that female virtual chatbots attracted more attentional resources and generated more emotional arousal than male virtual chatbots. A reasonable explanation is that the physical attributes of female virtual chatbots, owing to their good human nature (e.g., friendly, warm, and trusting), may be detected more easily than the physical attributes of male virtual chatbots (Borau et al., 2021). Previous studies have found that computers with female voices are perceived as more attractive (Lee et al., 2000), and recent research indicates that female systems elicit feelings of comfort, confidence, and reduced tension among users (Niculescu et al., 2010). Moreover, participants tended to perceive robots with a female body shape as more communal and having more cognitive and affective trust than those with a male body shape (Bernotat et al., 2021). These findings are in line with those of our study. Consequently, female virtual chatbots generated more positive emotions than male virtual chatbots. This result supported H1.

The data on female participants showed that female virtual chatbots evoked a higher amplitude of P200 than male virtual chatbots in occipital areas. This finding suggested that female participants allocated more attentional resources when interacting with virtual chatbots of the same gender. The result not only aligns with the similarity-attraction paradigm (Byrne, 1997) but also validates gender role identification. One plausible explanation is that when female participants interact with female virtual chatbots, they perceive an inherent consistency or similarity in gender, fostering emotional connection and trust. This emotional connection, in turn, makes female participants more likely to view female chatbots as approachable and engaging partners. Hence, the result suggested that female participants, by matching the gender role expectations of the female virtual chatbots, allocated more attentional resources and exhibited positive emotional responses toward female virtual chatbots. Thus, H3 was supported.

## 5 Conclusions

This study used ERP techniques to explore the neural mechanism of usage intentions on virtual chatbots' gender. The observed gender effects in human–machine communication appear to be present at the neural level, rather than being solely reflected in subjective questionnaire responses. The results of the subjective questionnaire revealed that users have a larger usage intention toward female virtual chatbots than male virtual chatbots, regardless of participants' gender. The result of the neural activity process was as follows: (1) Around 100 ms, an initial stage of visual perception occurred, with participants perceiving the physical attributes of the virtual chatbots' gender. Female virtual chatbots attracted more attentional resources than male virtual chatbots. Female participants allocated more attentional resources to same-gender partners, and male participants also exhibited greater attention toward same-gender partners. (2) Between 170 and 270 ms, an evaluation and judgment stage took place. The physical attributes of the virtual chatbots' gender continued to be detected. In comparison to male virtual chatbots, female virtual chatbots received greater attention, and the intensity of intention to use female virtual chatbots was assessed. Female participants allocated more attentional resources to female virtual chatbots; likewise, male participants allocated more attentional resources to male virtual chatbots. Our research findings provide a foundation for designing better virtual robot appearances. Designers can better understand users' neural activity regarding virtual robots' gender, which will enable them to create improved products.

There are some limitations in the present study that should be noted. First, it is essential to note that the EEG recordings may have been influenced by participants' emotion during the interaction with multi-style chatbots, even though two virtual chatbot groups with three stages of age were used to mitigate monotony during the EEG recording. Thus, future studies should investigate different ages of virtual chatbots. Second, we recruited college students with similar educational backgrounds and comparable ages. However, the similarity of our participants may limit the generalizability of our findings to individuals of different ages and educational backgrounds. Therefore, future studies should consider diversifying the sample to enhance the applicability of the results.

## Data availability statement

The raw data supporting the conclusions of this article will be made available by the authors, without undue reservation.

## Ethics statement

The studies involving humans were approved by the Ethics Committee of the Institute of Neuroscience and Cognitive Psychology of Anhui Polytechnic University (AHPU). The studies were conducted in accordance with the local legislation and institutional requirements. Written informed consent for participation in this study was provided by the participants' legal guardians/next of kin.

## Author contributions

YD: Conceptualization, Data curation, Funding acquisition, Methodology, Supervision, Validation, Writing – original draft, Writing – review & editing. RG: Conceptualization, Data curation, Investigation, Methodology, Validation, Writing – original draft, Writing – review & editing. WL: Data curation, Funding acquisition, Investigation, Methodology, Software, Validation, Writing – review & editing. WZ: Conceptualization, Funding acquisition, Investigation, Methodology, Validation, Writing – review & editing.
